# Acellular porcine heart matrices: whole organ decellularization with 3D-bioscaffold & vascular preservation

**Published:** 2017-03-15

**Authors:** Alice S. Ferng, Alana M. Connell, Katherine M. Marsh, Ning Qu, Annalisa O. Medina, Naing Bajaj, Daniel Palomares, Jessika Iwanski, Phat L. Tran, Kapil Lotun, Kitsie Johnson, Zain Khalpey

**Affiliations:** 1 *Department of Surgery, Division of Cardiothoracic Surgery, University of Arizona College of Medicine, Tucson, Arizona, United States*; 2 *Department of Physiological Sciences, University of Arizona College of Medicine, Tucson, Arizona, United States*; 3 *Department of Biomedical Engineering, University of Arizona College of Medicine, Tucson, Arizona, United States*; 4 *University of Arizona College of Medicine, Tucson, Arizona, United States*; 5 *Department of Internal Medicine, Division of Cardiology, University of Arizona College of Medicine, Tucson, Arizona, United States*; 6 *Banner, University Medical Center, Tucson, Arizona, United States*

**Keywords:** porcine, heart, decellularization, acellularization, organ, bioscaffold, vascular

## Abstract

Regenerative medicine, particularly decellularization-recellularization methods via whole-organ tissue engineering, has been increasingly studied due to the growing donor organ shortage. Though numerous decellularization protocols exist, the ideal decellularization protocol for optimal recellularization is unclear. This study was performed to optimize existing heart decellularization protocols and compare current methods using the detergents SDS (sodium dodecyl sulfate), Triton X-100, OGP (octyl β-D-glucopyranoside), and CHAPS (3-[(3-cholamidopropyl) dimethylammonio]-1-propanesulfonate) through retrograde aortic perfusion via aortic cannulation of a whole porcine heart. The goal of decellularization is to preserve extracellular matrix integrity and architecture, which was analyzed in this study through histology, microscopy, DNA analysis, hydroxyproline content analysis, materials analysis and angiography. Effective decellularization was determined by analyzing the tissue organization, geometry, and biological properties of the resultant extracellular matrix scaffold. Using these parameters, optimal decellularization was achieved between 90 and 120 mmHg pressure with 3% SDS as a detergent.

**Relevance for patients:** This study provides important information about whole heart decellularization, which will ultimately contribute to heart bioengineering.

## 1. Introduction

While heart transplantation is currently the definitive treatment for end-stage heart failure, the massive organ shortage has led to increased regenerative medicine and whole-organ tissue engineering research [1-4]. One approach to tissue engineering is the decellularization-recellularization method [5,6]. This method has been successful in regenerating skin, bladders, bone, kidneys [7], liver [8], vessels and lungs [9-11]; however, the same level of success has been much more difficult to achieve in organs with functional units, such as the heart [12-17]. The vascularization, high metabolic demand and the low regenerative potential of hearts all contribute to bioengineering difficulties [12,13]. However, the first bioengineered whole rat heart was reported in 2008, achieved using a perfusion decellularization method with subsequent recellularization [18]. The perfusion decellularization method involved cannulation of the ascending aorta with retrograde aortic perfusion using detergents [18]. More recently, an alternate decellularization approach using serial perfusion and agitation of hypo-tonic solution has been used in porcine hearts [19], though the retrograde aortic perfusion method is more standardized [20].

Regardless of the delivery method, the end-goal of decellularization is to produce a bioartificial scaffold that resembles the three-dimensional structure and mechanical properties of native heart tissue, while maintaining structure of the extra-cellular matrix (ECM) sufficient for cellular adhesion and growth. The ECM consists of a complex network of proteins, proteoglycans and glycosaminoglycans, and is the direct environment for cells during recellularization. Far from an inert scaffold, the role of ECM has been increasingly recognized in cell signaling, differentiation and tissue homeostasis [21]. Additionally, it is through membrane receptors known as integrins and mechanosensitive ion channels in the ECM by which cells perceive signals such as shear stress and tensile forces [22]. Decellularized heart ECM would ideally have minimal fiber “fraying,” as well as a total volume and size similar to the initial state of each respective heart used. The scaffold must also have similar tensile and biaxial strain characteristics as a normal healthy heart. Currently, numerous decellularization protocols exist in an attempt to reach these goals.

One of the variables that affect these quantitative outcomes during the decellularization process is pressure control. The study of this topic is limited. It has been shown that automating pressure during decellularization with digital pressure sensors improves whole heart decellularization [23], however the effects of pressures have not been studied despite the variations in pressure between protocols. The same holds true for total decellularization time. Few studies focus on the differences in detergent exposure times within non-toxic ranges, and most studies simply try to limit total exposure time [24]. Instead, the main differentiating factor between protocols has been the detergents or other decellularization agents used. The detergents used in the first successful rat heart decellularization were Triton-X and sodium dodecyl sulfate (SDS) [18]. The authors perfused heparinized PBS with adenosine for 15 minutes, 1% SDS for 12 hours, and 1% Triton X-100 in a retrograde fashion for 30 minutes with each step followed by rinsing with deionized water [18]. At a larger scale using porcine hearts via retrograde aortic perfusion, successive perfusates of 0.02% trypsin/0.05% EDTA, 3% Triton X-100, and 4% deoxycholate with PBS rinses between reagents also resulted in successful decellularization [25]. Other porcine heart decellularization protocols implemented similar methods [25,26], with a few additional detergents such as CHAPS (3-[(3-cholamidopropyl) dimethylammonio]-1-propanesulfonate) [27] used in heart valves and other organs [28,29]. All detergents have the potential to inflict damage on the ECM and disrupt the ultrastructure, by damaging the collagen and glycosaminoglycans for example [29]. In particular, CHAPS retained ECM and mechanical elasticity following lung decellularization fairly robustly, which made this a detergent of interest [27]. Another novel decellularization agent, OGP (octyl β-D-glucopyranoside), recently showed promising results compared to other decellularization agents in this regard, and with less cytotoxicity to porcine pericardium seen in other solutions [30]. This solution has not yet been used in whole heart decellularization, nor have tissues exposed to OGP been tested in recellularization.

In the present study, OGP was used along with another nonionic detergent (Triton-X 100), an ionic detergent (SDS), and a zwitterionic detergent (CHAPS) to decellularize whole porcine hearts. The effects of these detergents were studied at varying pressures to determine the minimum retrograde perfusion pressure necessary to perform a complete decellularization. Furthermore, our study aimed to investigate the mechanical differences between the decellularized matrices following various detergent treatments, and to ultimately optimize the methodology used for whole heart decellularization.

## 2. Materials and Methods

### 2.1 Harvest and surgical preparation of porcine hearts

Adult porcine hearts were obtained from healthy Yorkshire and Hampshire pigs at the Food Product and Safety Laboratory of the University of Arizona and processed immediately following procurement (Institutional Animal Care and Use Committee protocol #13-418). At the time of collection, each whole animal weighed 130 +/– 10 kg. Procured porcine hearts weighed from 350 to 500 g.

The heart was surgically excised at the great vessels and aorta from adjacent mediastinal attachments. Cannulation of the aorta was performed by using a cable tie to secure the aorta to a 3/8” cannula connected to the perfusion tubing. A total of 34 hearts were procured, and 7 hearts were excluded from the study due to errors in procurement, mechanical or computer errors. 3 hearts were treated with CHAPS, 3 hearts with OGP, and the rest with SDS/Triton X-100. Therefore a total of 27 porcine hearts were included.

### 2.2 Bioreactor apparatus

**Figure 1:**
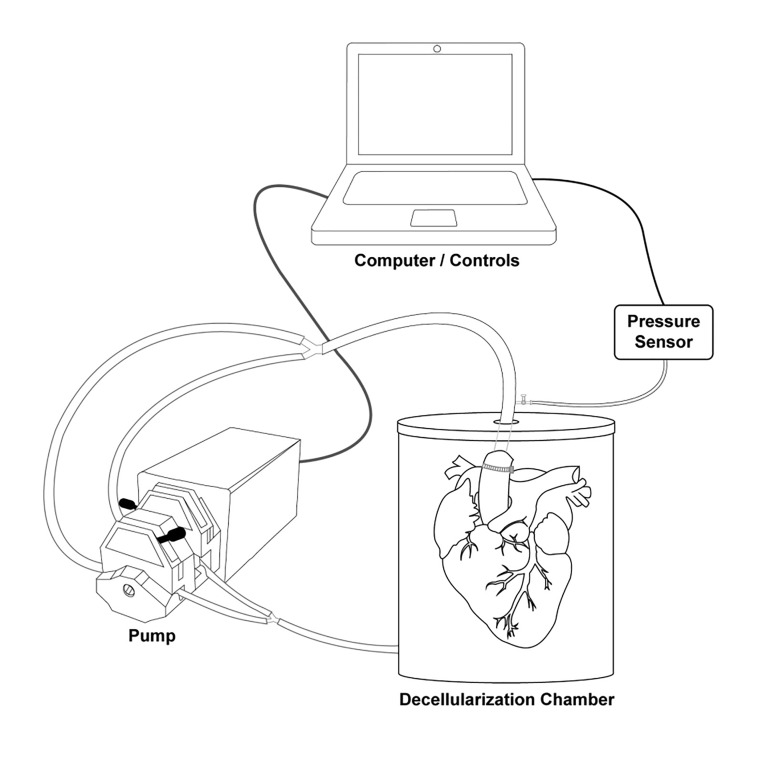
Decellularization apparatus and experimental setup.

The decellularization system used was a custom-built apparatus situated inside a laminar flow hood throughout the entire procedure (Figure 1). Peristaltic pumps were used to exchange solutions within the decellularization apparatus throughout the experiment. Digital pressure gauges and an analog manometer were used to determine the individual perfusion rates necessary to maintain continuous perfusion pressures of 70-80, 90, 120 or 140 mmHg per experimental condition. The manometer was monitored every hour for the first 36 hours, as the pressures vary the most between 0-36 hours, and at least once every 3 hours throughout the experiment to ensure pressures did not significantly vary after the first 36 hours.

### 2.3 Whole heart decellularization

The decellularization duration was determined by gross examination of the heart, histologic analyses, microscopic imaging, and mechanical testing. Retrograde aortic perfusion of the solutions took advantage of coronary perfusion while keeping pressure constant throughout the procedure with flow rates adjusted to remain around 90 mmHg, but under physiological pressures ≤120 mmHg. The aorta of each porcine heart was cannulated and remained in the customized chamber described above at room temperature within a cell culture hood for the entire duration of the procedure. All solutions were introduced via retrograde aortic perfusion through a series of pumps and carboys containing autoclaved, sterile-filtered solutions. Constant pressure was maintained at 70-80, 90, 120 or 140 mmHg for exploratory experiments, with ideal flow rates remaining around 90 mmHg.

A computer is used to adjust pump settings based on the readings from the pressure sensor. This is connected directly to the forward flow of reagents and detergents into the heart from the pump.

The decellularization solutions used were: 3%, 5%, and 10% SDS, CHAPS, and 1% OGP, and 3% Triton X-100. All solutions were autoclaved or filtered. Decellularization with SDS involved use of Triton X-100 to aid in removal of residual SDS, and subsequent PBS to remove Triton X-100. Following decellularization, the heart was washed with diH_2_O and 1X PBS. The optimal decellularization using SDS is as follows: (1) 45 mins: heparin rinse with 10,000 U/L, (2) repeat step 1, (3) 10 mins: diH_2_O rinse, (4) 12 hours: 3% SDS, (5) 10 minutes: diH_2_O rinse, (6) 24 hours: fresh 3% SDS, (7) 10 minutes: diH_2_O rinse, (8) 24 hours: 3% Triton X-100, (9) 10 mins: 1X PBS rinse, (10) repeat step 9 twice more, (11) 24 hours: 1X PBS, (12) 24 hours 1X PBS. This leads to a total decellularization time of approximately 110 hours. Decellularization with CHAPS and OGP was performed as a single detergent experiment, with the same amount of time in detergent as SDS/Triton X-100 experiments. Table 1 lists the experimental conditions tested.

### 2.4 Histological assessment

Each decellularized and native heart was biopsied at the right and left atria, right and left ventricle, and right and left auricles for morphological analysis. The samples were fixed overnight at room temperature with 10% neutral-buffered formalin, embedded in paraffin, and sectioned into 10-micron thick adjacent sections. Hematoxylin and eosin (H&E) staining was used to evaluate the presence of nuclear material by standard light microscopy on a Leica microscope. Masson’s trichome stain was used also to evaluate the collagen in the decellularized heart ECM.

### 2.5 Transmission electron microscopy (TEM)

From both decellularized and native hearts, 5-mm^3^ specimens were cut for processing and analysis. Samples were fixed in2.5% glutaraldehyde in PIPES buffer (pH 7.4) overnight. Samples were washed three times for 10 minutes with PIPES, fixed in 1% osmium tetroxide and then followed by two washes in DI water. Samples were stained in a block with 2% uranyl acetate and dehydrated through a graded ethanol series (50%, 70%, 90%, and 100%). Following infiltration with Spurr’s Resin, the blocks were polymerized at 60°C overnight. 70 nm sections were cut on a Leica EMUC6 ultra microtome onto150 mesh copper grids. Sections were stained with 2% lead citrate and viewed in an FEI Tecnai Spirit electron microscope operated at 100 kV. 8 bit TIFF images were collected via an AMT 4 megapixel camera.

**Table 1:** Experimental conditions for heart decellularization.

Experimental condition	Decellularization method used	Result summary
70-80 mmHg	SDS + Triton-100	Incomplete: Pressures were not high enough for complete decellularization after 5 days.
90 mmHg	SDS + Triton-100	Complete: This was the ideal pressure to use, and resulted in complete decellularization.
120 mmHg	SDS + Triton-100; CHAPS; OGP	Complete: While the results were within reason using SDS, the matrix at 90 mmHg more closely resembled native cardiac tissue. CHAPS and OGP showed an incomplete decellularization after 5 days, and even waiting 21 days total using fresh solutions, decellularinzation was still incomplete.
140 mmHg	SDS + Triton-100; CHAPS; OGP	Complete: Even though full decellularization was seen in SDS hearts, this pressure was too high, and resulted in matrix that had wide spacing. At this pressure, CHAPS and OGP did not result in a fully decellularized heart.

All experiments listed were performed at constant pressure, with an n = 3 for each condition.

### 2.6 Scanning electron microscopy (SEM)

From both decellularized and native hearts, 5-mm^3^ specimens were cut for processing and analysis, using a standardized protocol. The 3% SDS method was used for the decellularized tissue samples. The specimens were first fixed with 2.5% glutaraldehyde in PBS (pH 7.4) at 4°C overnight and washed with DI water. Samples were then dehydrated in a graded ethanol series (50%, 70%, 90%, and 100%) v/v in DI water at 10-minute intervals for each concentration. Following this, specimens were freeze-dried with liquid CO_2_ using a critical point drying apparatus (Polaron model 3100, Energy Beam Sciences, East Granby, CT). Samples were mounted on aluminum stubs with carbon double-sided tape and sputter-coated with a 5-nm-thin layer of gold (Pelco SC4, Ted Pella, Inc., Redding, CA) to provide surficial conduction.

### 2.7 DNA quantification

Tissue samples were excised from the same anatomical areas in both native and decellularized hearts (e.g., right atrial epicardium or left ventricular endocardium), and wet tissue weight was used for normalization prior to DNA quantification. Approximately 100 mg of native and decellularized porcine hearts were incubated with 400 µl cell lysis buffer and 8 µl Proteinase K (Viagen, 20 mg/ml) overnight in a 55°C water bath. Samples were then placed in 90°C for 10 minutes to ensure inactivation of Proteinase K. The digest was vortexed for 1 minute and centrifuged at 13,000 rpm for 10 minutes. The supernatant was transferred to a new tube and the DNA was precipitated with isopropanol and centrifuged to pellet the DNA. The DNA pellet was rinsed with 70% ethanol, dried, and re-suspended in nuclease-free water. DNA was quantified using a Thermo Scientific NanoDrop^™^ 1000 spectrophotometer. The DNA concentration of each chamber of the heart was measured, along with the left and right auricles. Tissue samples were separated into the epicardial and endocardial layers. DNA analysis was performed for each sample, and in this case, 3 separate experiments using the same protocol was performed, and the DNA concentration results were averaged.

### 2.8 Mechanical testing of cardiac tissue

The dynamic mechanical behavior of decellularized left ventricular myocardial tissue was measured with a Perkin-Elmer Pyris Diamond dynamic mechanical analyzer (DMA) through sinusoidal oscillation of rectangular specimens in tension. The tissue was cut into rectangular bars of width 10 mm, thickness 1 mm, and length ~20 mm. The rate of oscillation was 250 μm/min, with a duration of 20 minutes and an endpoint of 5000 μm, or until the sample slipped from the harness. Prior to each run, the DMA was calibrated by adjusting the force read by the DMA with a 50 g standard weight. Characterization was performed in 1X PBS at 37°C, at a pH of 7.4. Strain was calculated as the % change in length (m), which was plotted against the corresponding stress (kPa). Young’s modulus for elasticity was determined from the initial, linear section of the plotted traces.

### 2.9 Assessment of collagen content

A hydroxylproline assay kit (Sigma Aldrich, MAK008-1KT) was used to assess the content of insoluble collagen in cardiac tissue samples. The wet weight of each sample was used for normalization prior to assays. The manufacturer’s protocol was used for the assay, and absorbance readings were taken at an excitation wavelength of 560 nm.

### 2.10 Angiogram of decellularized heart

Decellularized hearts were imaged using a Toshiba mobile C-arm (Surginix SXT-2000A) system. Images were obtained in the cranial, caudal, RAO and LAO planes. Perfusion of the coronary sinus was imaged using a 14Fr Edwards Lifesciences^™^ Retrograde Cardioplegia Catheter (Edward Lifesciences Services GmbH, Germany) with Isovue-300 (Iopamidol Injection 61%, molecular weight: 777.08 g/mol) for contrast media (Bracco Diagnostics Inc, BIPSO GmbH, Germany). Subsequent fluoroscopy images were taken of the left and right main coronaries using a 2.1 mm right angle Coronary Artery Perfusion Cannula with a self-inflating 5.0 mm balloon (Vitalcor, Westmont Illinois). A 25 G *PrecisionGlide* Needle (Becton Dickinson & CO, Franklin Lakes NJ) was used for epicardial injections into the apex of the left ventricle. Digital angiography videos were obtained for each injection route. The optimized 3% SDS decellularization method was used for these hearts at a flow rate of roughly 120 mmHg.

## 3. Results

### 3.1 Decellularization of whole porcine heart

**Figure 2:**
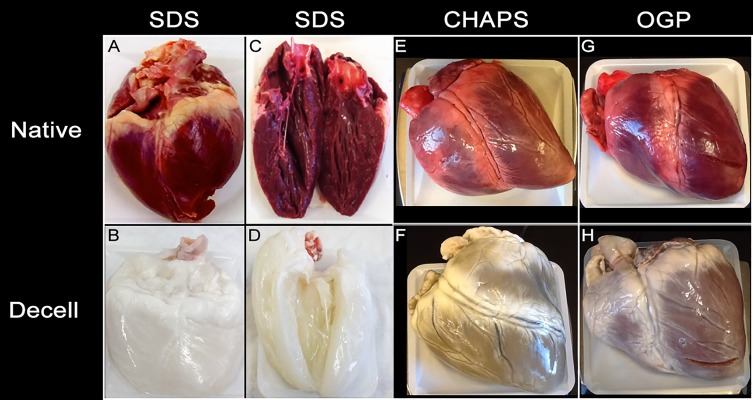
Gross images of the native control porcine heart (A, C, E, G) and decellularized porcine heart (B, D, F, H) before and after experiment. C and D are cut in the sagittal plane. As labeled, panels B and D show a visually fully decellularized heart using SDS. Following the same protocol, CHAPS (F) and OGP (H) resulted in a heart that was not fully decellularized via gross inspection.

**Figure 3:**
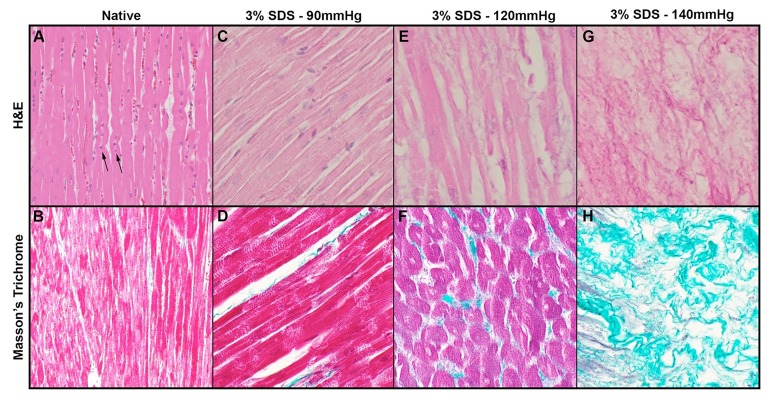
Histological images of the native and decellularized heart under selected perfusion pressures. As found from experimentation, 3% SDS decellularization methods were ideal over other detergents, and the same protocol was used for each set of hearts (n=3) at 90, 120, and 140 mmHg. Under standard H&E staining, nuclei (denoted by arrows) in the native heart (A) can be seen, while trichrome staining demonstrates native collagen meshwork of the native heart (B). The authors report that perfusion pressures between 90 and 120 mmHg are ideal for decellularization. At 90 mmHg, it can be seen that while some nuclei remain, they are not viable (C), while the collagen structure of the heart is preserved (D). Under 120 mmHg of perfusion pressure, nuclei are no longer present in the tissue (E), however, the collagen meshwork has become looser and less compact in structure (F). At 140 mmHg, even though all nuclei and other cellular components appear to be fully removed (G), the normal collagen meshwork and structure of the native heart is no longer intact and is not recognizable as cardiac tissue (H). Images taken at 10x magnification.

**Figure 4:**
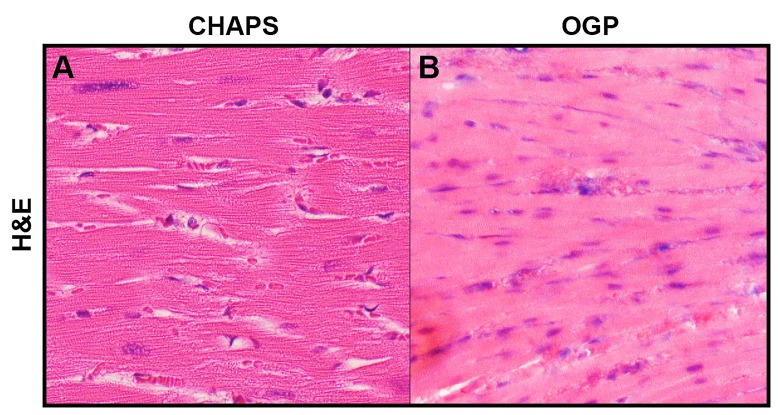
Histological images of decellularized heart experiments using CHAPS and OGP. Using standard H&E staining, nuclei are found to be present in left ventricular tissue removed from hearts that have undergone decellularization using detergents CHAPS and OGP. Native cardiac structure is well-preserved while some nuclei remain viable after treatment with CHAPS (A). Similarly, nuclei are also still viable and present after treatment with OGP (B), therefore demonstrating that this detergent also cannot be used for whole organ decellularization of porcine hearts. Images taken at 10x magnification.

The ultimate objective of organ decellularization is to remove all of the cellular material without adversely affecting the composition, biologic activity, or structural integrity of the remaining three-dimensional extracellular matrix. In this study, decellularization conditions were controlled using a customized bioreactor system. 3% SDS with subsequent Triton X-100 resulted in a successful decellularized heart. By gross observation of the surface of the heart (Figure 2A) and with a sagittal cut (Figure 2C), decellularization appeared successful (Figure 2B and 2D, respectfully). As expected, each heart also had a decreased weight after decellularization (pre-decellularization weight: 431 ± 54 g; post-decellularization weight: 320 ± 70 g; average ± SD). The results from the exploratory experiments performed at constant pressures of 70-80, 90, 120 or 140 mmHg, separately, are summarized in Table 1. Histological data of decellularization with 3% SDS at 90, 120, and 140 mmHg is shown in Figure 3. By gross visualization, CHAPS (Figure 2F) and OGP (Figure 2H) treated hearts were not successfully decellularized; this was further confirmed by histology of OGP and CHAPS (Figure 4) where nuclei remain preserved in the collagen matrix and decellularization is incomplete.

### 3.2 Histological assessment

H&E staining revealed no viable basophilic staining representative of cellular nuclear material in the left ventricle after 5 days using the SDS decellularization protocol at 90 mmHg (Figure 3C and 3D). In contrast, cell nuclei were visibly viable in the left ventricle of the native control heart (Figure 3A and 3B), as evidenced by the presence of basophilic staining. The tissue morphology remained largely intact following decellularization, as native and decellularized cardiac histology can be directly compared, with cell ghosting seen in the decellularized heart where cardiomyocytes are present in native cardiac tissue or non-viable nuclei (Figure 2C). Cardiac architecture after tri-chrome collagen staining of decellularized samples (Figure 3D, 3F, 3H) was representative of native hearts at pressures ≤ 120 mmHg (Figure 3B), but did demonstrate loose organization of myocytes and more collagen bundles as pressures increased past 120 mmHg. At 120 mmHg, nuclei are no longer present nor viable (Figure 3E) and collagen meshwork is less dense (Figure 3F). At 140 mmHg, while nuclei and cellular components are completely removed (Figure 3G), there is little semblance of the decellularized collagen matrix to the structure of native heart tissue (Figure 3H). Decellularization with CHAPS or OGP shows both presence of viable nuclei with a preserved collagen matrix, although this indicates an incomplete decellularization (Figure 4).

### 3.3 Scanning electron microscopy (SEM)

**Figure 5:**
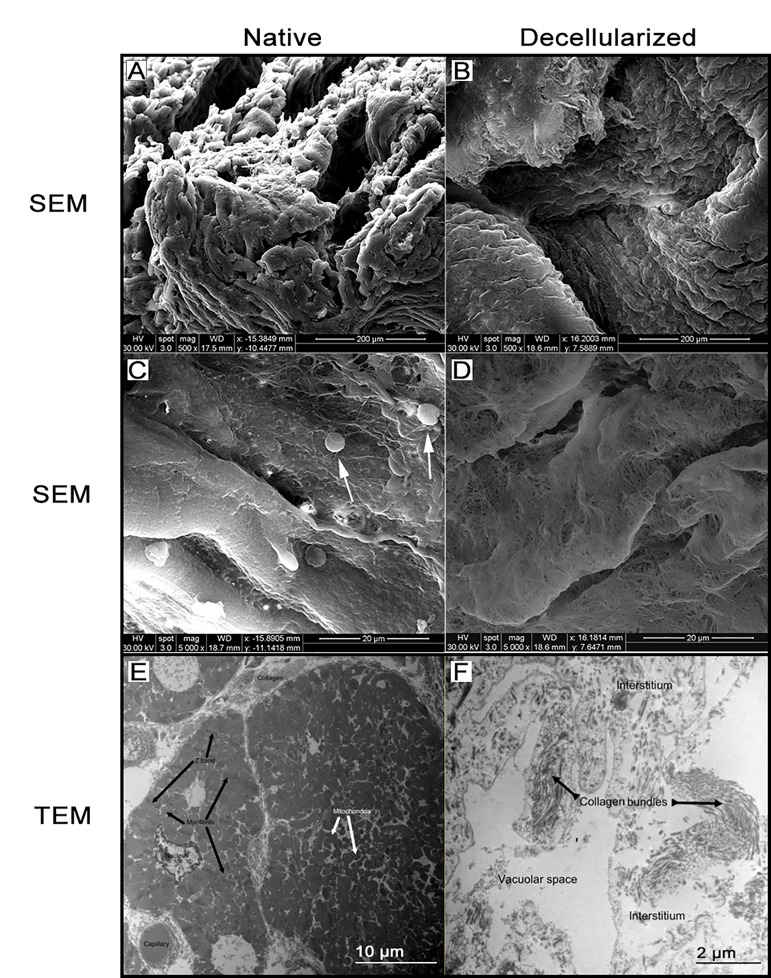
Scanning electron microscopy (SEM) images of native and decellularized porcine heart. Fibrillar meshwork of the porcine heart was similar between the native and decellularized tissues (A vs. B), with no clear loss of junctional meshwork following decellularization. The removal of cells from the tissue surface can be seen when comparing native heart (C; white arrows point to cells) to decellularized heart (D). Structural similarities regarding fiber structure, pore size, and tertiary matrix structures can be seen between native (A, C) and decellularized (B, D) samples, suggesting that integrity of the tissue and collagen structure has been maintained following decellularization. Transmission electron microscopy (TEM) images of native (E) and decellularized (F) porcine heart. There was an absence of cellular structures, such as mitochondria, from the decellularized cardiac tissue. However, collagen bundles were preserved in the decellularized cardiac tissue.

SEM analysis (Figure 5) indicated the maintenance of architecture after intact porcine heart 5-day decellularization using 3% SDS. The epicardial left ventricular wall of the porcine heart in the native control (Figure 5A) was comparable to the epicardial wall of decellularized heart tissue (Figure 5B). This shows that there was architectural preservation following the 5-day decellularization process. The endocardial surface of the decellularized heart (Figure 5D) indicated a topographic variance and intact ECM fibers without the presence of cells (shown by arrows in Figure 5C), which showed that ECM components, particularly collagen, were preserved.

### 3.4 Transmission electron microscopy (TEM)

TEM imaging indicated that there were no nuclei at the completion of the 5-day decellularization process (Figure 5F). However, tissue basement membrane was intact and architectural integrity was largely maintained while cellular material has been predominantly removed since collagen bundles were still present and clearly recognizable on TEM (Figure 5F). In the native heart tissue, cellular components such as the mitochondria were clearly visualized (Figure 5E).

### 3.5 DNA quantification

**Figure 6:**
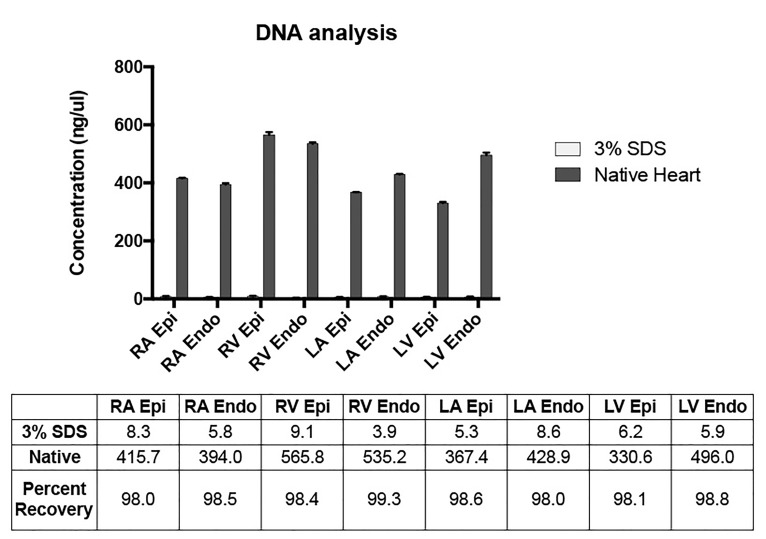
DNA analyses of each chamber of the heart following decellularization. RA = right atrium; RV = right ventricle; LA = left atrium; LV = left ventricle; epi = epicardium; endo = endocardium. Values listed in the rows for 3% SDS and Native refer to concentrations of DNA in ng/ul. Percent recovery represents the DNA content that was removed from decellularized tissue samples as compared to native heart tissue.

DNA quantification analysis from native and decellularized hearts demonstrated a significant decrease in the amount of DNA present in decellularized heart tissue compared to native heart tissue (Figure 6). Using a NanoDrop spectrophotometer, it was determined that over 90% of DNA was removed in SDS decellularized tissue from each chamber of the heart via a constant perfusion pressure of 120 mmHg, prior to the use of nucleases for recellularization.

### 3.6 Cardiac tissue mechanics

The epicardial layer of the left ventricle from each respective experiment was stretched via sinusoidal oscillation at 37°C until the tissue tore apart or slipped out of the machine. Young’s modulus of elasticity was then calculated from the slope of the initial linear portion of each data plot collected (Table 2). The native heart demonstrated the largest modulus of elasticity (271.8 ± 55.6) when compared to the decellularized heart, suggesting the native hearts possessed the most resistance and least elasticity. Hearts decellularized with 3% SDS exhibited an elastic modulus most similar to the native heart (272.4 ± 26.4 vs. 272.4 ± 26.4, *p* = 0.49). On the contrary, the elasticity of the heart decellularized with 5% SDS (217.1 ± 28.5) was significantly higher than the native heart or the heart decellularized with 3% SDS. The latter trend contin ued for hearts decellularized with 10% SDS (28.8 ± 0.7), CHAPS (3.6 ± 0.8), and OGP (1.2 ± 0.3), respectively.

**Table 2:** Dynamic materials analysis of cardiac tissue.

Experimental Condition	Young’s Modulus (kPa)
Native Heart	271.8 ± 55.6
Decell with 3% SDS	272.4 ± 26.4
Decell with 5% SDS*	217.1 ± 28.5
Decell with 10% SDS*	28.8 ± 0.7
Decell with CHAPS*	3.6 ± 0.8
Decell with OGP*	1.2 ± 0.3

The myocardial layer of the left ventricle was stretched via sinusoidal oscillation at 37°C. Young’s modulus of elasticity was calculated, and values are representative from 3 consecutive runs of samples in each experimental group due to limited tissue availability. Mean ± standard error of mean. *p < 0.05 with respect to the native heart.

### 3.7 Assessment of collagen content

**Figure 7:**
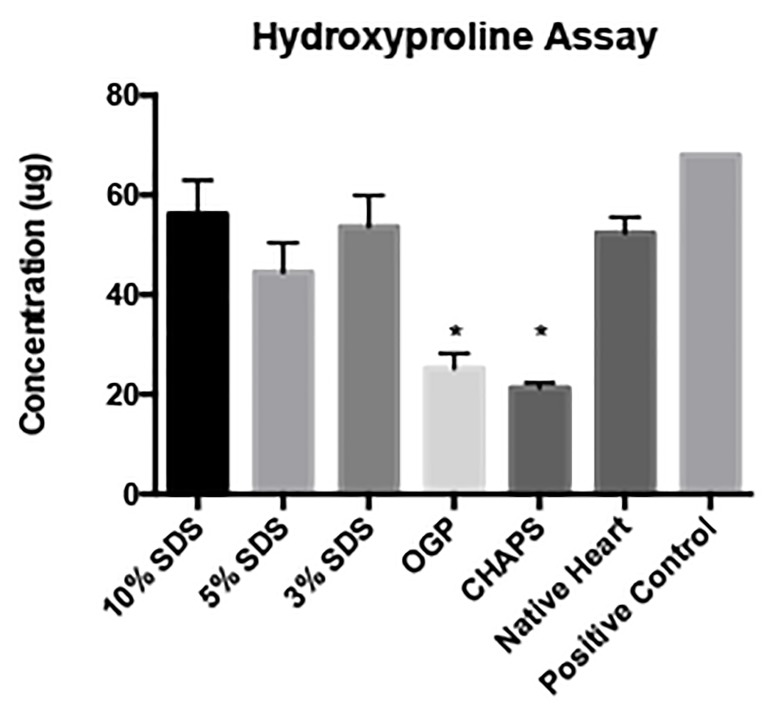
Hydroxyproline assay for collagen content. The collagen content of the hearts decellularized with SDS was similar to the native heart. OGP and CHAPS had significantly less collagen content than the native heart. n=3 for each group; * = *p* < 0.05; error bars expressed as SEM

The collagen content of the hearts decellularized with SDS was similar to the collagen content found in the native heart (Figure 7). OGP and CHAPS had significantly less collagen content than the native heart.

### 3.8 Angiogram of decellularized heart

**Figure 8:**
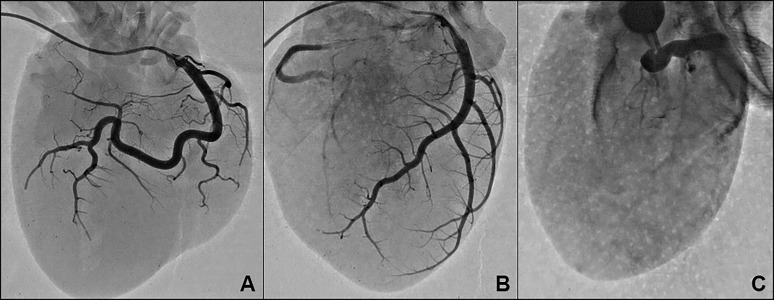
Angiogram of Decellularized Heart. The angiogram shows patency and vascular integrity of the vessels through the right coronary artery (A), left coronary artery (B), and coronary sinus (C). The optimized 3% SDS decellularization method was used for these hearts.

The results of the angiogram through the right coronary artery, left coronary artery, coronary sinus, as well as the associated branches and circumflex arteries can be seen in Figure 8, showing patency of the vasculature.

## 4. Discussion

Despite many years of research on the topic of decellularization, a multitude of difficulties remain. The replicability of decellularized bio-scaffolds using existing methods remains inconsistent, calling for a decellularization standard [31]. In an attempt to quantitatively standardize decellularization results, Crapo *et al.* reviewed general parameters with an emphasis on minimizing residual DNA. Though decellularization techniques have thus far been unable to remove 100% of cell material in larger animal model and human hearts [14-16], these parameters all focus on limiting nucleic material since residual DNA is directly correlated to adverse host reactions and may contribute to cytocompatibility issues upon reintroduction of cells [31,32]. Prior to the use of nucleases to remove DNA, we were interested in the effectiveness of retrograde perfusion through the aorta and wanted to identify which regions had higher residual DNA content. In line with the established parameters, the decellularized porcine hearts in the present experiment had < 50 ng DNA per mg ECM dry weight from each of the 4 heart chambers (Figure 6) and lacked visible nuclear material in tissue sections stained with H&E (Figure 3). The final DNA content in the endocardium and epicardium of the right ventricle was greater than that of the left ventricle, whereas the right and left atria had comparable DNA concentrations. The differences in the DNA content of the ventricles might be explained in part by the use of retrograde aortic per-fusion, defined this way due to the retrograde flow through the aortic cannula. Perfusion in this manner inevitably causes differences in perfusion pressures across regions of the heart, with left heart pressures greater than the right. This therefore results in variation of decellularization across the heart chambers and vasculature.

Another general complication in the field of decellularization is “scaling up” current methods to human-sized organs. Much of the existing research involves the use of rodent organs, which are orders of magnitude smaller than a porcine heart. The larger overall size and increased heart wall thickness pose a challenge to achieving full decellularization of porcine at low pressures. Using a pressure of 70 mmHg resulted in incomplete decellularizations, whereas a pressure of 140 mmHg disturbed the ECM integrity (Table 1, Figure 3G and 3H). To note, the formalin used in H&E preparation also likely affected ECM integrity. However, the same preparation was used across experimental and control groups in order to control for these potential differences. After testing various perfusion pressures between 70-140 mmHg, it was determined that a minimum perfusion pressure > 80 mmHg but < 120 mmHg was optimal for achieving complete decellularization through a whole porcine heart. As observed for 3% SDS at 90 mmHg, the nuclei of decellularized tissue is non-viable, although preserved in the collagen matrix (Figure 3C and 3D).

Whereas at 120 mmHg, the nuclei are non-viable and more completely removed, while the collagen meshwork is now looser (Figure 3E and 3F). It is unknown how loose the collagen meshwork can be before recellularization efforts become unsuccessful. Looser meshwork requires more extracellular matrix to be laid, and while introduced cells (stem cells, fibro-blasts, etc.) could attach to decellularized tissue, they may be too spaced apart for effective intracellular signaling to trigger recellularization, regeneration, and proliferation of cardiac tissues and cell types. Maintaining a truly constant perfusion pressure was not possible due to the fact that flow rates change depending on the amount of material removed from the native heart, as well as the viscosity of the fluids both newly introduced and over time. We used a digital pressure sensor (PendoTECH, Princeton, NJ) set to adjust the flow rate to create a constant perfusion pressure, which was checked by a manual manometer. Due to the flux of viscoelasticity of the heart ECM and remaining blood/cellular contents over the duration of decellularization, there was a variability of 10-20 mmHg in perfusion pressure across experiments. Our proposed decellularization method results a complete decellularization determined by histology (Figure 3-4), microscopy (Figure 5), and DNA analysis (Figures 6). Moreover, a complete decellularization can be grossly determined by a translucent-white tissue appearance (Figure 2), as opposed to maintaining native tissue coloring. Finally, an angiogram performed on a heart decellularized with 3% SDS showed that the vasculature was intact and patent through the right coronary artery, left coronary artery, coronary sinus, circumflex arteries, and associated branches (Figure 8).

Beyond the DNA, histological and microscopic analyses, as well as the gross appearance and intact vasculature, the final engineered heart tissue must have the structural integrity to develop systolic force following recellularization. It must be sufficiently compliant to withstand physiological diastolic loads, and form an electromechanical syncytium. Hence, it is important to preserve the structural and matrix components of the heart, consisting of collagen, elastin, proteoglycans, glycosaminoglycans, fibronectin, and laminin, among other components. A hydroxyproline assay was done to analyze insoluble collagen content on decellularization experiments performed with 10% SDS, 5% SDS, 3% SDS, OGP, and CHAPS (Figure 7). The collagen content of the hearts decellularized with SDS was not significantly different than native heart. OGP and CHAPS had significantly less collagen content than the native heart.

SDS hearts had similar positive findings in measurements of tissue elasticity, evaluated by recording the maximum force necessary to tear the outside layer of the heart. With systolic function in mind, dynamic materials analysis was performed on left ventricular myocardial tissue and the elasticity modulus was calculated for the native heart and decellularization experiments performed with 10% SDS, 5% SDS, 3% SDS, OGP, and CHAPS (Table 2). Native cardiac tissue had the highest elastic modulus, followed by 3% SDS, 5% SDS, 10% SDS, CHAPS, and OGP, respectively. While we tested a series of detergent concentrations, it was determined that 3% SDS was the minimum concentration required to perform a complete decellularization within our 5-day experiment. Greater amounts of detergents had a degenerative effect on the ECM, demonstrated by the lower elasticity values. Since use of CHAPS and OGP produced incompletely decellularized hearts grossly (Figure 2F, H) and histologically (Figure 4), it was determined that optimal conditions could not be produced and therefore no further analysis needed to be performed on these detergents. To try and determine if this suboptimal decellularization with CHAPS and OGP was due to the shorter protocol for SDS, CHAPS and OGP experiments were repeated and continued for a total of 21 days, including PBS and water washes. However, even the extended time did not produce optimal results. Cellular components remained even after lengthening the usual 5-day method to 21 days at a constant pressure of 120 mmHg when using CHAPS and OGP. A higher perfusion pressure of 140 mmHg produced similar results for CHAPS and OGP treated hearts. We therefore focused on optimizing our results with the SDS/Triton X-100 method.

In summary, this study aimed to build upon existing heart decellularization protocols to further optimize and standardize current methods. Perfusion pressures were added as an additional experimental variable and mechanical tissue properties as an additional endpoint. To optimize reseeding potential of a decellularized bioscaffold, we propose using retrograde aortic perfusion via aortic cannulation, between 90 and 120 mmHg pressure, and 3% SDS as a detergent for optimal decellularization.
